# Revolutionizing Phenolic Content Determination in Vegetable Oils: A Cutting-Edge Approach Using Smartphone-Based Image Analysis

**DOI:** 10.3390/foods13111700

**Published:** 2024-05-29

**Authors:** Sanita Vucane, Ingmars Cinkmanis, Karina Juhnevica-Radenkova, Martins Sabovics

**Affiliations:** 1 Food Institute, Faculty of Agriculture and Food Technology, Latvia University of Life Sciences and Technologies, LV-3004 Jelgava, Latvia; ingmars.cinkmanis@lbtu.lv (I.C.); martins.sabovics@lbtu.lv (M.S.); 2 Processing and Biochemistry Department, Institute of Horticulture, LV-3701 Dobele, Latvia

**Keywords:** total phenols, vegetable oils, digital image analysis, RGB color model, smartphone

## Abstract

This study addressed the need for a more accessible and efficient method of analyzing phenolic content in vegetable oils. The research aimed to develop a method that could be widely adopted by both researchers and industry professionals, ultimately revolutionizing the way phenolic content in vegetable oils is analyzed. This study developed a method of determining the total phenolic content (TPC) in vegetable oils using smartphone image analysis in the RGB color model. The method employed a gallic acid calibration solution and demonstrated exceptional determination coefficients for the RGB colors. The R—red color was selected as the basis for the analyses, and the method was statistically equivalent to standard UV/Vis spectrophotometry. The highest TPC was determined in hemp and olive oils, while the lowest was found in rice bran, grapeseed, and macadamia nut oils. This study concluded that smartphone image analysis, mainly using the R component of the RGB color model, was a superior alternative to traditional spectrophotometric methods for determining the TPC in vegetable oils. This innovative approach could revolutionize phenolic content analysis by providing researchers and industry professionals with a cost-effective, safe, and efficient tool. The estimated limit of detection (LOD) of 1.254 mg L^−1^ and limit of quantification (LOQ) of 3.801 mg L^−1^ further confirmed the reliability and comparability of the method. With these findings, it was expected that the method would be widely adopted in the future.

## 1. Introduction

Vegetable oils are a natural product derived from various plants, primarily consisting of triacylglycerols [1]. The unsaturated fatty acids within triacylglycerols are vulnerable to oxidation when exposed to oxygen, heat, light, and other catalysts. This oxidative process can result in the development of off-flavors, odors, and the degradation of the nutritional quality in oils and fats [2]. Vegetable oils are susceptible to oxidation due to the formation of free radicals and other reactive oxygen species (ROS), which have the potential to pose a risk to human health by causing oxidative stress and inducing damage at the cellular and molecular levels [3]. In addition to triacylglycerols, vegetable oils may contain biologically active substances, such as phenolic compounds [2]. The presence of phenolic compounds in oil can influence its oxidative stability and, consequently, its shelf-life [4]. Phenolic compounds serve as natural antioxidants, aiding in the prevention of oxidation and rancidity in the oil [5]. This, in turn, can help extend the oil’s shelf-life and preserve its nutritional and sensory quality over time. The quantity of phenolic compounds can be utilized as a criterion to evaluate the overall quality of oil and as an indicator for stability prediction [6]. As they naturally occur in plants, phenolic compounds exhibit a wide range of health-promoting properties due to their biological activity [7]. Their identification allows for differentiation between various types and brands of vegetable oils based on their antioxidant properties, which can be a valuable marketing point for consumers seeking healthier options. Given the aforementioned observation and the extensive utilization of oil in the food, cosmetics, and pharmaceutical industries [8,9], it is crucial to determine the presence of phenolic compounds in vegetable oils.

The determination of the total phenolic content (TPC) in vegetable oils can be achieved through various analytical techniques. These include spectrophotometry [10], high-performance liquid chromatography (HPLC) [11], gas chromatography [12], and colorimetric assays [13]. Each method has its own advantages and should be selected based on the specific requirements of the analysis, the nature of the phenolic compounds present, and the available resources. One of the most commonly used methods for determining TPC in vegetable oils is the spectrophotometric assay, also known as the Folin–Ciocalteu method [14]. This method is preferred for its simplicity, cost-effectiveness, and widespread availability, making it suitable for quick estimations of TPC in various samples [15,16]. However, while spectrophotometric analysis is simple, it does require an initial investment to cover the costs of the spectrophotometer and the necessary reagents for the analysis. Moreover, spectrophotometric analysis typically requires a stable laboratory setting, limiting its use to a stationary environment. This lack of mobility may be a drawback for researchers or analysts who need to perform on-site or field measurements, where portability and flexibility are essential. In such cases, alternative methods or portable analytical devices may be more suitable for determining the TPC in vegetable oils. Lastly, spectrophotometric methods often lack the sensitivity to reliably assess the limit of detection (LOD) and limit of quantification (LOQ) for certain phenolic compounds in vegetable oils. This limitation can make it challenging to accurately detect and quantify low concentrations of phenolic compounds, especially in complex matrices where interferences may be present [17]. As a result, researchers may need to explore alternative analytical techniques with lower LOD and LOQ values to ensure the accurate and reliable quantification of TPC in vegetable oils.

Owing to technological progress, smartphones are increasingly gaining popularity and accessibility as an alternative to chemical analysis within the food and medical sectors [17]. The use of smartphones in chemical analysis offers several notable advantages. Firstly, their portability enables their use in various locations beyond the confines of a laboratory environment [18]. Moreover, and secondly, their cost-effectiveness provides a potentially more economical solution when compared to the equipment for traditional chemical analysis [19]. Thirdly, the elimination of extensive instrument maintenance and calibration requirements significantly reduces expenses. Finally, the user-friendly nature of smartphones allows for ease of use without the need for specialized knowledge [20]. Smartphones can complement the conventional analysis equipment and methods [21]. With the growing interest in smartphones for chemical analysis, this approach can aid in examining the TPC of vegetable oils, similar to smartphone-based methods developed for estimating phenolics of a polar nature in aqueous and alcohol-derived extracts [22,23,24,25,26,27,28,29]. In addition to the analysis of the TPC, the applicability of a smartphone for the study of antioxidant activity, i.e., radical cation scavenging against 2,2′-azino-bis(3-ethylbenzothiazoline-6-sulfonic acid) (ABTS) and radical scavenging against 1,1-diphenyl-2-picrylhydrazyl (DPPH) of food products has been demonstrated [30]. Overall, the outcomes obtained through the application of smartphone-based methods are comparable and as accurate as those achieved through conventional analysis methods such as spectrophotometers. However, based on the available literature reported so far, smartphone-based methods for analyzing TPC were performed for aqueous extracts, and only one scientific manuscript has indicated the use of smartphone image analysis to determine the TPC in vegetable oils, specifically olive oil [31]. However, verifying the smartphone-based method and its applicability for plant-derived oil samples would be necessary to ensure accuracy and specificity. Moreover, the calculated limits of detection (LOD) and quantification (LOQ) of the analyses demonstrated relative variations.

After carefully considering these limitations, the present research focused on investigating the feasibility of determining the TPC in vegetable oils using smartphone-based image processing and comparing the results with those achieved through the conventional spectrophotometry method.

## 2. Materials and Methods

The method involved the utilization of the Folin–Ciocalteu phenol reagent, which is a commonly used chemical reagent for detecting the presence of phenolic compounds in various plant-based samples. In this case, it was used to determine the TPC in vegetable oils. The analysis was performed by capturing images of the samples using a smartphone camera, where the Red–Green–Blue (RGB) color model was used to analyze the images. This was completed in a photo studio Puluz Box, Shenzhen Puluz Technology Ltd., (Shenzhen, China), which provided a controlled environment for capturing accurate and consistent images.

### 2.1. Materials

#### 2.1.1. Samples

For the purpose of TPC analysis, a total of eleven different vegetable oils were selected. These oils included sea buckthorn, sunflower, rice bran, macadamia nut, hemp, corn, grapeseed, linseed, rapeseed, olive, and milk thistle. All of these oils were obtained in their original commercial packaging to ensure the accuracy and consistency of the analysis.

#### 2.1.2. Equipment Used for Total Phenolic Content Analysis

The equipment used included the following:

The Agilent Cary 60 UV/VIS spectrophotometer (Agilent Technologies, Inc., Palo Alto, CA, USA).

The photo studio Puluz Box (Puluz Technology Ltd., Shenzhen, China) featured an open front and the following specifications: material: polyvinyl chloride; number of LED lamps: 40 pcs; LED lamp model: 2835; lamp brightness: 24–26 lumens; white light color temperature: 6500 Kelvins (K); warm white color temperature: 3200 K; power: 4–8 W; voltage: 5 V; indication: Ra > 82; sizes: outer diameter 120 mm, inner diameter 86 mm; power supply: USB port.

For the research, the best TechRadar in the Mobile Choice Consumer Awards (MCCA), winner of the TechRadar Mobile Choice Consumer Awards (MCCA) in 2019 was chosen from the Chinese electronics manufacturer Huawei with the P smartphone series P30 Lite (Table 1).

### 2.2. Methods

#### 2.2.1. Smartphone Image Acquisition

**Figure 1:**
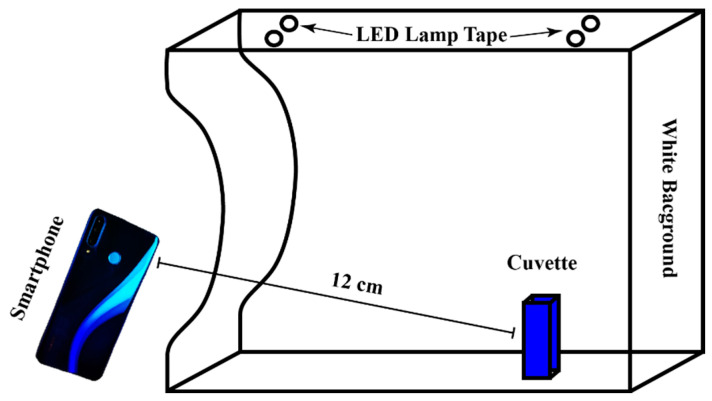
Illustration of photo studio and smartphone placement for colorimetric imaging.

A Huawei P30 Lite smartphone (Huawei Technologies Co., Ltd., Shenzhen, China) was placed horizontally in front of the open-side photo studio Puluz enclosure 12 cm, with 2.5 mL disposable polystyrene macro cuvettes (BrandTech™ Scientific, Inc., Essex, CT, USA), with 96% ethanol (blank), gallic acid (standard), or vegetable oil samples with Folin–Ciocalteu reagent. Colorimetric analysis was performed according to Figure 1 on a white background.

#### 2.2.2. Image Analysis

The images of oil were captured with the use of a smartphone camera (Android), utilizing the RGB color model. The “ColorMeter” color picker application version 3.1.1 (VisTech Projects, Atlanta, GA, USA), which is available on Google Play store (version 41.0.28-29 [0] [PR] 633720010), was used for this purpose. The resulting image was saved in 8-bit JPG format with a size of 7.0 (8000 × 6000 pixels), and phase detection autofocus (PDAF), 27 mm (wide), ISO 400 (placeholder), f/1.8 (aperture of the camera lens), 1/2.0 (size of the camera sensor), and 0.8 μm (pixel size of the camera sensor) were used in order to produce the desired outcome.

#### 2.2.3. Preparation of Calibration Curves for Total Phenolic Content Determination

A gallic acid standard, obtained from Sigma-Aldrich Chemie Ltd. (Steinheim, Germany), was prepared at varying concentrations ranging from 1 to 200 mg L^−1^ in a 96% ethanol solution. To measure the concentration of each standard, 0.5 mL of the solution was mixed with 2.5 mL of 0.2 M Folin–Ciocalteu phenol reagent (Sigma-Aldrich). After a 5 min incubation period, 2 mL of 7.5% Na_2_CO_3_ solution (Sigma-Aldrich) was added to the mixture. The mixture was then allowed to incubate for 2 h at room temperature (21 ± 1 °C), after which the absorbance was measured at 760 nm using a spectrophotometer and a smartphone. A reference solution of 96% ethanol was used in the spectrophotometer and smartphone.

**Figure 2:**
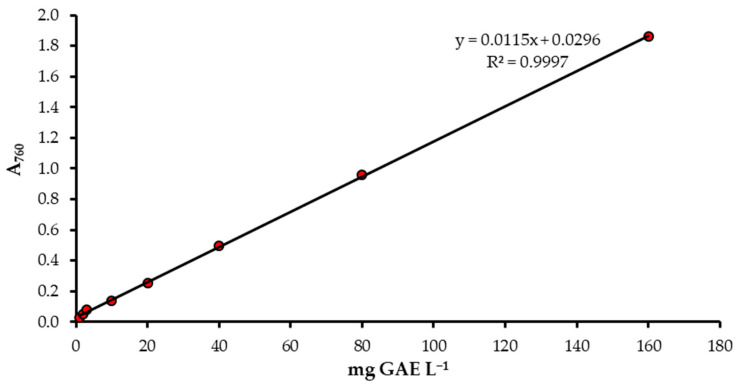
Calibration curve of gallic acid (mg GAE L^−1^) with spectrophotometer.

The gallic acid (mg GAE L^−1^) calibration curve using a spectrophotometer is shown in Figure 2.

#### 2.2.4. Determination of Total Phenolic Content in Vegetable Oils

Briefly, 0.5 g of vegetable oil was mixed with 2.5 mL of 0.2 M Folin–Ciocalteu reagent and stirred for 1 min with an IKA Vortex 3. After 5 min, 2 mL of 7.5% Na_2_CO_3_ solution (Sigma-Aldrich) was added to 15 mL in a 120 × 17 mm conical polypropylene tube (Sarstedt AG & Co.KG, Lower Saxony, Germany) and stirred for 30 s with an IKA Vortex 3 (IKA^®^-Werke GmbH & Co. KG, Staufen, Germany) at speed 7. After 2 h incubation at room temperature (21 ± 1 °C), the emulsion was centrifuged at 10,000 rpm for 5 min. The colored solution was transferred to 2.5 mL PS disposable macro cuvettes (BrandTech™ Scientific, Inc., Essex, CT, USA) with dimensions of 12.5 × 12.5 × 45 mm. The absorbance of the sample against a reference solution (96% ethanol) was measured at 760 nm using an Agilent Cary 60 UV/VIS spectrophotometer. The detection of oil samples with Folin–Ciocalteu phenol reagent and 96% ethanol solution in cuvettes was performed with images taken using a smartphone according to the illustration in Figure 1. The analyses were performed in 10 replicates.

Analysis of acquired images and calculations: The TPC was calculated using a gallic acid calibration curve and the results were expressed as the equivalent of gallic acid mg 100 g^−1^ oil (mg GAE 100 g^−1^).

(1) Color values were calculated for the images obtained from the smartphone application “ColorMeter” using the RGB color model for the following colors: for calibration solution—Red (R_avg_), Green (G_avg_), and Blue (B_avg_), and for vegetable oils—Red (R_avg_) according to Equations (1)–(3), as follows:

(1) $$R_{avg}=\frac{\sum_{i=1}^{n}R_{i}}{n}$$

(2) $$G_{avg}=\frac{\sum_{i=1}^{n}G_{i}}{n}$$

(3) $$B_{avg}=\frac{\sum_{i=1}^{n}B_{i}}{n}$$

(2) The average color value of the calibration solution (R_avg_, G_avg_ and B_avg_) and vegetable oils (R_avg_) was converted to absorbance using the modified Beer–Lambert (Equation (4)) [32], as follows:

(4) $$Abs=-\log\left(\frac{I}{I_{0}}\right)$$

where: 

I—averaged value of R_avg_, G_avg_ or B_avg_ and

I_0_—average value of R, G, or B color of 96% ethanol solution.

**Figure 3:**
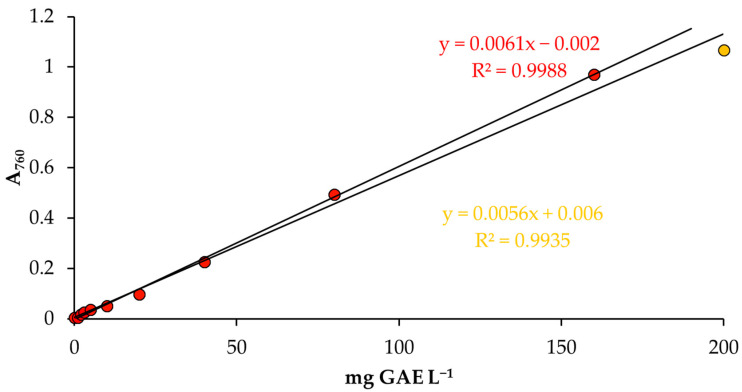
Gallic acid calibration curve was obtained using red (R) color intensity as the basis for analysis by smartphone. The linearity of the gallic acid calibration was achieved within the concentration range of 1 to 160 mg L^−1^ (represented by red dots) and from 1 to 200 mg L^−1^ (represented by yellow dots).

(3) Calculation of total phenolic content (TPC) concentration from the light intensity of the red color (C_R(TPC)_) was performed according to Figure 3, followed by Equation (5)).

(5) $$C_{R(TPC)}=\frac{{(Abs}_{R}+0.002)}{0.0061}=mg\;GAE\;100\;g^{-1}\;oil$$

A Python algorithm was developed to calculate the TPC from the smartphone images and retrieve the RGB color module data. This algorithm can be utilized on Microsoft Windows 10, 11, macOS, Linux, or web browser (Kaggle (Web App) [33] environments and performed the analyses using the high-level programming language Python. The complete and comprehensive code of this algorithm, which was used to determine the TPC from analyzing the smartphone images, is available on GitHub: https://github.com/ (accessed on 14 May 2024) (Project name: Analysis of smartphone images to determine TPC) [34].

## 3. Results and Discussion

### 3.1. Determining the Optimal Distance for Image Acquisition

**Figure 4:**
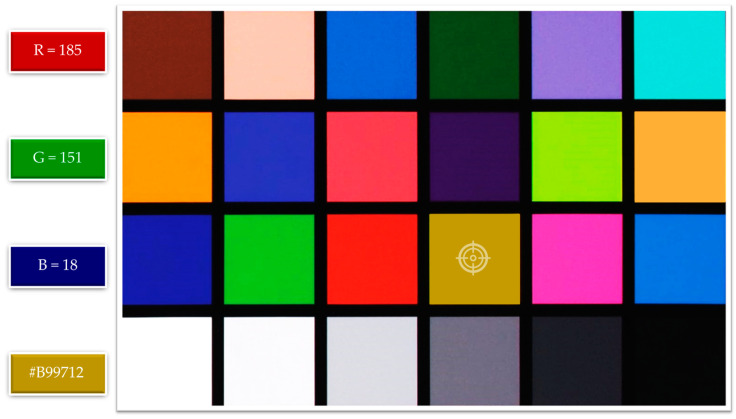
Image of the X-Rite ColorChecker color panel.

**Figure 5:**
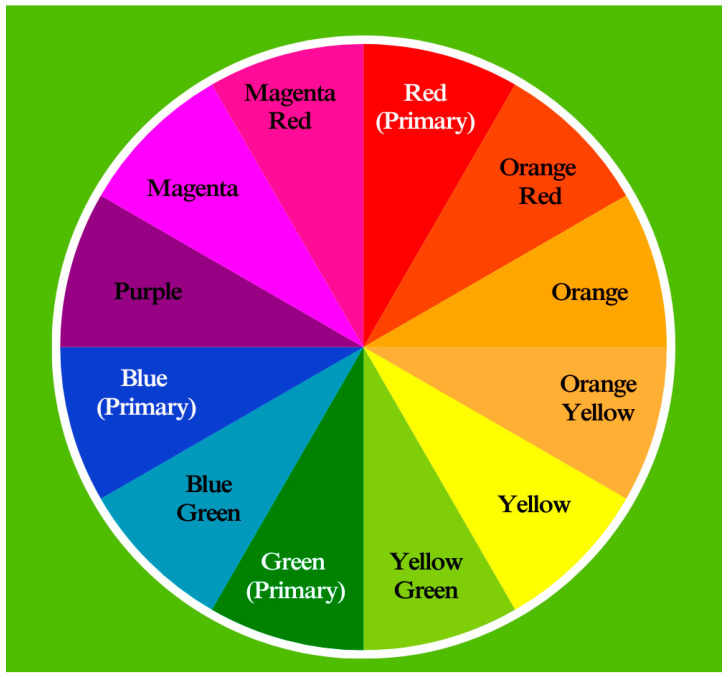
Color wheel of primary and secondary colors.

When performing the analysis of the TPC in vegetable oils using smartphone-based image capturing, it is necessary to determine the optimal distance from the smartphone camera lens to the object to acquire quality images and accurate indicators of the RGB color model. The importance of distance between the lens and the object is crucial during the analysis of phenolics as it directly impacts the focus, clarity, and resolution of the images captured, affecting the reliability of the analysis results, as in Calabria et al. [35]. Maintaining the appropriate distance ensures the accurate and precise measurements of the TPC in the sample. According to this, the analyzed oils were filled in 2.5 mL disposable macro cuvettes made of polystyrene and placed in a photo studio box. The distance was determined using the X-Rite ColorChecker color panel, a standard for calibrating colors and balances for professional cameras for image and video capture. The yellow color was detected as R = 185, G = 151, and B = 18 pixels in the RGB color model (185, 151, 18), using the yellow color as a reference for determining the optimal distance (Figure 4). This was performed because blue is the primary color for determining the TPC, and it is essential to understand how distance affects complementary colors, which, in the case of oils, is primarily yellow, or primary red (R), and green (G) (Figure 5). 

**Figure 6:**
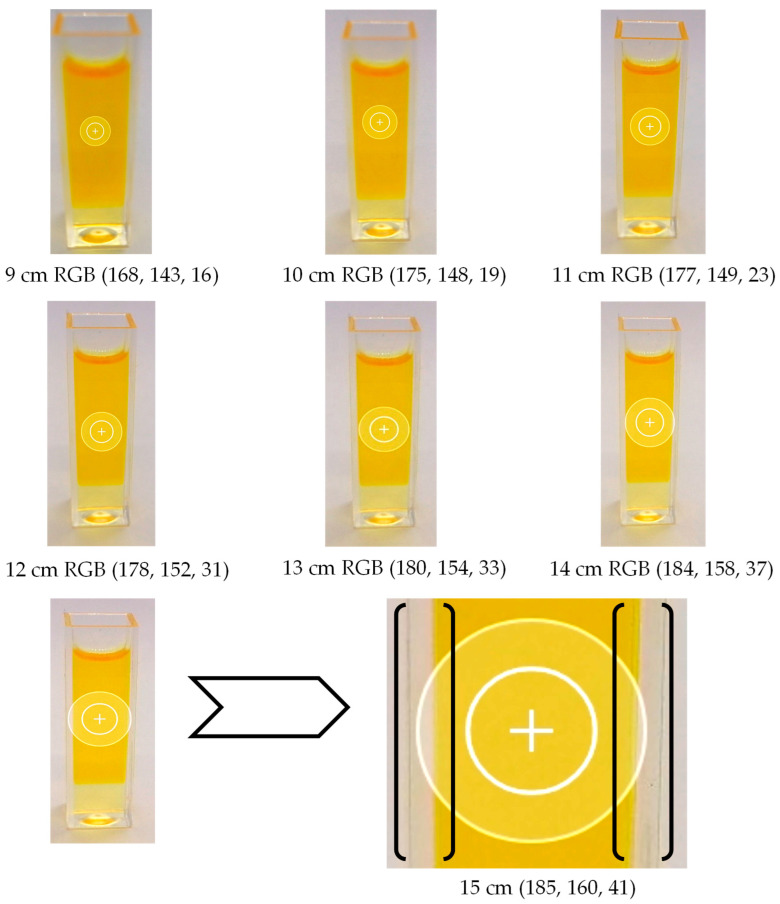
Rapeseed oil color images, RGB color model values, and detection regions obtained by setting the smartphone at a certain distance.

The specified color is analyzed against the X-Rite ColorChecker color panel for yellow to determine the optimal RGB color model values for rapeseed oil, aiming for RGB 185, 151, 18 pixels. Upon evaluating the resulting images, the closest optimal distance to the X-Rite ColorChecker color panel is found to be 12 cm with RGB 178, 152, and 31. While the red (R) component improves with increasing distance, the green (G) and blue (B) spectra tend toward the white color area, reaching a value of 255 pixels. The pixel values increase as the distance exceeds 12 cm, indicating a tendency of the RGB color model toward white. As shown in Figure 6, the range of the detection region changes depending on the smartphone’s distance, which affects the numeric values of the RGB color model. The calculated *t*-values (*p* < 0.001) for color were significantly higher than the critical one, affirming the precision of the research finding. 

With a 95% confidence level, we can confidently assert that there are significant statistical differences between the distances from the smartphone lens, the analyzed object, and the values of the RGB color model. As illustrated in Figure 6, the boundaries of the 15 cm detection region fall outside the area of the rapeseed oil cuvettes. In the process of analysis, the calculation of the white background color is considered alongside the yellow color, contributing to the pursuit of the maximum RGB color value of 255 pixels. As the distance decreases below 12 cm, there is an observable shift towards the black color, or 0 pixels, in contrast to the white color, indicating a decrease in the intensity of the yellow color tone. In the study of Anh-Dao et al. [17], changing the distance from the camera to the cuvette did not significantly affect measured intensities. However, the precision varied, with a 10 cm distance being the most favorable. The authors recommended a 10 cm distance for the smartphone camera and noted the need to establish calibration curves for each smartphone due to potential variations in the obtained RGB values. The pursuit of a black tone can be explained by the reduction in light between the smartphone lens and the object to be analyzed, which does not allow the light generated by the LED diodes to pass through the vegetable oil during the absorption process, and is facilitated by the poor focusing of the lens, thus creating a blurred image. Minh-Huy et al. [19] documented a relatively shorter distance between the lens and the object, indicating the ability to capture clear images at a distance of 8 mm from the camera. Additionally, the authors emphasized the significance of LED light intensities within the custom colorimetric analysis box for accurate result acquisition.

The X-Rite ColorChecker color panel, a reliable tool in color analysis, was used to determine how the specified distance affects the black or white color. The obtained data, presented in Table 2, further validate the robustness of the proposed methodology.

The *t*-values calculated for the colors white (3.69, *p* > 0.05) and black (0.12, *p* > 0.05) were found to be below the critical value. Therefore, at a 95% confidence level, it can be stated that there is no significant statistical difference between the distances from the smartphone lens and the X-Rite ColorChecker color panel for black and white. This implies that the distance between 9 and 15 cm does not affect the change in the white or black color, as there are no obstacles for the light to reach the lens. The change in color is mainly influenced by the object being studied, which absorbs a certain amount of light, or by the boundaries of the detection region being outside the area of the cuvettes of an analyzed object. It is also important to consider the color of the background used, as this can cause errors in obtaining the true color. Minh-Huy et al. [19] highlighted the significance of the background in their work, emphasizing its substantial impact on the accuracy of smartphone-based methodology. The highest level of precision in the results was attained when utilizing a white background.

### 3.2. Determination of Total Phenolic Content

**Figure 7:**
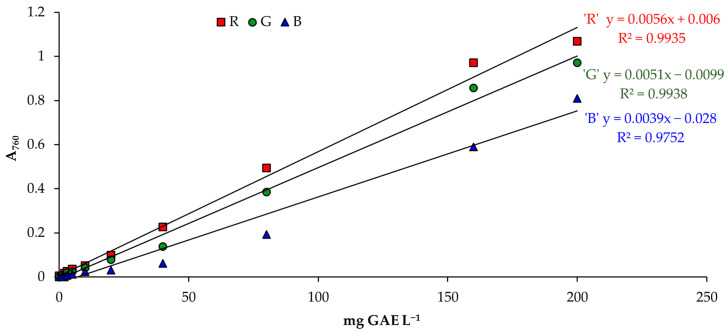
Gallic acid calibration curve using RGB color model obtained by smartphone.

Upon analysis of the RGB color model of the image in question, it was determined that a high coefficient of determination can be achieved for the calibration solution of gallic acid across all three color channels, namely R, G, and B. The coefficient of determination was found to be highest for the R channel (*R*^2^ = 0.9935), followed closely by the G channel (*R*^2^ = 0.9938), and the B channel (*R*^2^ = 0.9752), as shown in Figure 7. These results indicate that the RGB color model can be reliably utilized for detecting and quantifying the concentration of gallic acid in the solution.

It was found that the red color (R) provides the best detection factor when evaluating the validation data. However, a better determination coefficient (*R*^2^ = 0.9988 (*p* > 0.05)) can be obtained with the equation y = 0.0061x − 0.002 if the concentration of standard gallic acid is up to 160 mg L^−1^ (Figure 3). This result is similar to the conventional spectrophotometry method (*R*^2^ = 0.9997 (*p* > 0.05)) (Figure 2).

Although the green (G) and blue (B) coefficient of determination of the color spectrum is close to that obtained by the spectrophotometer method, the red (R) color intensity was used as the basis for determining the content of the TPC. The intensity of the red color shows a better coefficient of determination as explained in the color circle (Figure 5). In the Folin–Ciocalteu method, the determination of the TPC is based on the difference in the formation of the blue color intensity from the amount of phenol compound. The color circle shows that the complementary color of blue is orange. When using the “ColorMeter” Android application on a smartphone to take images, only three primary colors can be displayed—red (R), green (G), and blue (B). Therefore, to determine the content of common TPC, it is necessary to choose the primary color that is closest to the complementary or secondary color for orange. The closest primary color to orange in this circle is red.

A conventional UV/Vis spectrophotometer was employed to compare the images obtained using a smartphone. Upon analyzing the data obtained from the spectrophotometer and the smartphone, it was observed that the differences in the TPC are only within the range of ±0.007 to 0.21 mg GAE 100 g^−1^ of oil. The *t*-value obtained for the red—R color spectrum results of the smartphone image analysis was below the critical value (0.47, *p* > 0.05), thus indicating that there is no statistically significant difference between the TPC obtained using the two methods at the 95% confidence level.

**Figure 8:**
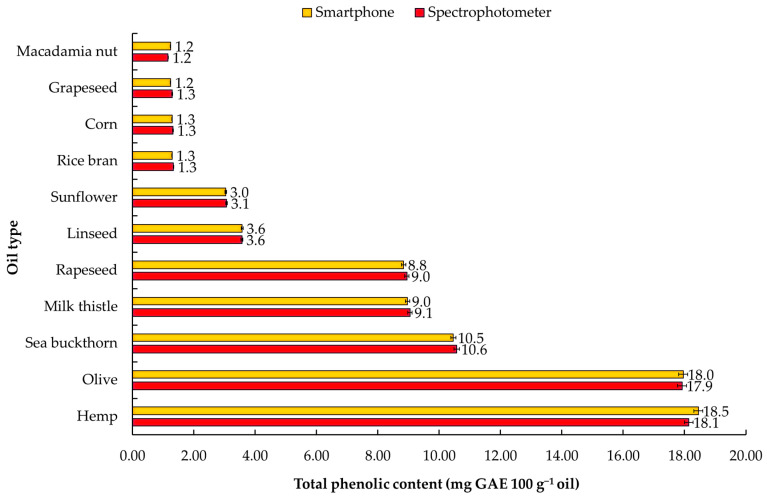
Total phenolic content (TPC), expressed as mg of gallic acid equivalents (GAE) per 100 g^−1^ of oil.

The determination of TPC is an important aspect of assessing the quality and health benefits of vegetable oils. In this study, the concentration of TPC was measured in various vegetable oils using both spectrophotometry and smartphone image analysis methods (Figure 8). The highest concentrations of TPC were found in hemp and olive oils, with values of 18.0 ± 0.2 and 17.9 ± 0.2 mg GAE 100 g^−1^ oil, respectively, detected by spectrophotometry. Smartphone image analysis also ensured comparable results, with values of 18.4 ± 0.2 and 17.9 ± 0.2 mg GAE 100 g^−1^ for hemp and olive oil, respectively. Notably, there is a paucity of literature on the use of smartphone image analysis to quantify the TPC of vegetable oils. However, the observed TPC values utilizing spectrophotometer are consistent with those reported by Kalinowska et al. [23], indicating the TPC range from 0.4 to 15.4 mg GAE 100 g^−1^ oil.

Additionally, these data can be supported by observations made by Mikołajczak et al. [2], revealing the range of TPC in hemp oil from 2.45 to 267.5 mg GAE 100 g^−1^. The rice bran, grapeseed, and macadamia nut oils exhibited the lowest concentrations of TPC using both methods, with values ranging from 1.2 to 1.3 mg GAE 100 g^−1^ oil. The results are consistent with those reported in [2,36,37,38]. According to the scientific evidence, the low TPC in rice bran, grapeseed, and macadamia nut oils is conditioned by the hydrophilic nature of phenolics, which limits the solubility of bioactives in the oil [39]. It has been indicated that these plant materials are represented mostly by phenolic acids, flavanols, and flavan-3-ols, such as procyanidin B1, trans-resveratrol, epicatechin, and catechin, ferulic and coumaric acids, quercetin, and kaempferol documented so far to be sparingly soluble in oils [40,41,42]. In turn, hemp is known to contain a variety of phenolic compounds, including flavonoids, stilbenes, and phenolic acids. Some of the main phenolics found in hemp include cannflavins A and B, cannabichromene (CBC), and cannabidiol (CBD). These compounds have been shown to have potential health benefits, such as anti-inflammatory and antioxidant properties [43,44]. The solubility of the phenolic compounds found in hemp can vary depending on the specific compound. Generally, cannabinoids like CBD are not very soluble in water, but are highly soluble in organic solvents like ethanol and oil-based solvents [45]. Considering the content of TPC in macadamia nut oil, the results are inconsistent with those reported by Shuai et al. [46], indicating the range of TPC from 77.9 to 123.4 mg GAE 100 g^−1^. The high TPC content is due to the extraction method used, where methanol was used as a solvent for extracting bioactive additives.

As indicated, the relative standard deviation (%RSD) for both spectrophotometry and smartphone colorimetry was in the range of 0.1 to 4.8%. The concentration limit of TPC varies widely in the literature sources, which may be attributed to several factors such as the type of vegetable oil, degree of ripening, regional and climatic conditions, harvesting period, type of extraction, storage, and packaging.

### 3.3. Validation of a Smartphone Analytical Method for Total Phenolic Content

Determining the pixel values of the red (R) color image obtained on the smartphone was used to obtain the absorbance results of gallic acid concentration. The following equations were obtained: y = 0.0061x − 0.002 (*R^2^* = 0.9988) and y = 0.0056x + 0.006 (*R^2^* = 0.9935), evaluating the linearity of gallic acid calibration in the range from 1 to 160 and from 1 to 200 mg L^−1^, respectively (Figure 3). An enhanced linearity was observed when employing a 9-point calibration curve, which encompassed gallic acid concentrations ranging from 1 to 160 mg GAE L^−1^.

Based on the statistical analysis (Table 3) of the comparison of both calibration curves for the two concentration ranges, it was calculated that the slope (a) for the 1 to 160 mg L^−1^ (0.00608) is slightly higher than for the 1 to 200 mg L^−1^ range (0.00563). The intercepts (*b*) are close to zero for both ranges, demonstrating minimal baseline offset. The slope error (*a* error) for the 160 mg L^−1^ (0.00007) range is significantly lower than that for 200 mg L^−1^ (0.00015), indicative of a more precise slope estimation. Similarly, the intercept error (*b* error) is lower for the narrower range (0.004 compared to 0.012), suggesting less intercept variability. The *F*-value for the 1 to 160 mg L^−1^ range (6778.851302) is considerably higher than that for the 1 to 200 mg L^−1^ range (1375.24616), suggesting a significantly better model fit and less error variance for the narrower range. Degrees of freedom (*df*): The degrees of freedom are 9 and 8 for the ranges 1 to 200 mg L^−1^ and 1 to 160 mg L^−1^, respectively, representing the number of data points minus the number of estimated parameters slope and intercept.

The regression sum of squares for the range 1 to 160 mg L^−1^ (0.879735097) is smaller than that for the range 1 to 200 mg L^−1^ (1.56657465), indicating less variability in the data explained by the model. The difference in the sum of squares is significantly smaller in the range 1 to 160 mg L^−1^ (0.00103821) compared to the range 1 to 200 mg L^−1^ (0.010252108), indicating better fit and less error in the narrower range model. The *p*-value for the *F*-test is much smaller in the range 1 to 160 mg L^−1^ (5.28141 × 10^−13^) compared to the range 1 to 200 mg L^−1^ (3.7374 × 10^−11^), which shows a very significant difference in the quality of fit between the two ranges.

**Figure 9:**
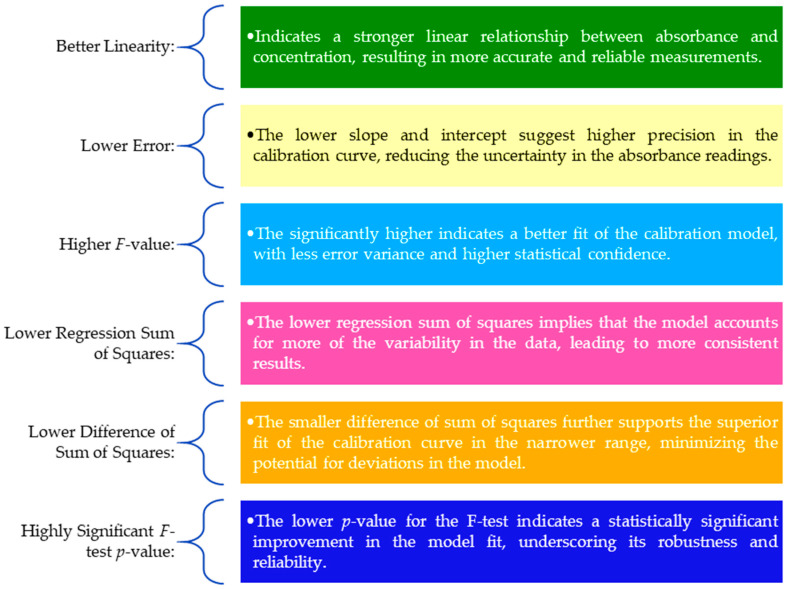
Major key factors of the calibration curve for the range 1 to 160 mg L^−1^ compared to the range 1 to 200 mg L^−1^ of gallic acid.

The calibration curve for the range 1 to 160 mg L^−1^ is better than the range 1 to 200 mg L^−1^ due to several key factors explained in Figure 9.

Based on the statistical analysis of the *F*-test (Table 3) and the linear nature of the obtained curve, it has been determined that utilizing the gallic acid concentration within the range of 1 to 160 mg L^−1^ in the 9-point calibration would lead to more precise outcomes (Table 4).

A novel method for detecting the TPC in vegetable oil has been developed utilizing a digital image captured on a smartphone, based solely on red light from the RGB color model. The method has a limit of detection (LOD) of 1.254 mg L^−1^ and a limit of quantification (LOQ) of 3.801 mg L^−1^. The method has a measurement range of gallic acid that spans from 3.801 to 160 mg L^−1^. The LOD and LOQ values reported in the study by Anh-Dao et al. [18] support the findings, showing a LOD range of 1.20 to 2.20 mg GAE L^−1^ and a LOQ range of 3.60 to 6.60 mg GAE L^−1^ for the determination of TPC in coffee products. The authors suggest using the B channel for plant extracts rich in TPC and the R channel for those with a lower TPC content.

The accuracy and repeatability of the method were assessed by subjecting each sample of vegetable oil to analysis in 10 replicates. The TPC of the vegetable oil samples was determined, resulting in a relative standard deviation (%RSD) of 0.67% (Table 5). Minh-Huy et al. [19] achieved a similar level of precision in their smartphone-based TPC determination, with %RSDs ranging from 0.92% to 5.8% on a white background and from 5.5% to 49% on a black background. The use of a white background is recommended as it reduces light reflection during LED illumination inside the custom colorimetric analysis box. Similar precision (%RSD < 2.4) was achieved by Lucas et al. [28], indicating the reliability and accuracy (98.4 to 103.8%) of the developed smartphone-based method for analyzing TPC in plant extracts using the HSV color model, specifically the S color parameter.

It is noted that the %RSD for spectrophotometry reported in the literature varies between 0.1 and 4.8% [18,47,48,49]. However, the %RSD for the method developed in this study was notably lower. The lower %RSD reinforces the high precision of the method, indicating minimal variability in the measurements relative to the mean value, and thus establishes the method’s reliability and consistency in producing results. The average repeatability of the method was calculated to be 0.0344, with a maximum of 0.1414 mg GAE 100 g^−1^ of oil. These values demonstrate relatively low variation in repeated measurements, signifying positive accuracy. The low average repeatability underscores the method’s consistent results with minimal deviations. However, the maximum value highlights an occasional higher variability, which, although infrequent, should be duly noted. The average accuracy for vegetable oils is 0.0689 mg GAE 100 g^−1^ of oil (Table 5), representing the average deviation of the measured values from the actual value in several vegetable oil samples. An average accuracy close to zero indicates consistent results very close to the actual value, suggesting that the average measurements for this amount slightly deviate from the actual value. Although this deviation is slight, it is crucial to consider the measurement range and analysis conditions. The maximum accuracy value of 0.2828 mg GAE 100 g^−1^ of vegetable oils represents the most significant observed deviation from the actual value between measurements. While such deviations may occur, their presence underscores the potential for considerable deviation under certain circumstances. Various factors, such as sample heterogeneity, matrix effects, instrumental limitations, or procedural errors, can contribute to this. The results of the present study provide evidence of the reliability and robustness of the method. In summary, smartphone-based analysis of TPC can be applicable in various fields such as food quality assessment [50], environmental monitoring [51], and agricultural research [52]. By utilizing the computational capabilities and optical sensors of smartphones, this approach enables the rapid and cost-effective analysis of phenolic compounds in various samples. It has the potential to facilitate on-site testing, remote monitoring, and data collection in resource-limited settings, thereby enhancing the accessibility and efficiency of phenolic compound analysis. Additionally, it can empower researchers, farmers, and food industry professionals to make timely decisions based on real-time phenolic compound data.

## 4. Conclusions

The RGB color model has been found to be a reliable and efficient method for quantifying total phenolic content (TPC) in vegetable oils when analyzing smartphone images. This study showed that the coefficient of determination for the red color spectrum closely aligns with traditional UV/Vis spectrophotometry, unlike the green and blue spectra. This innovative approach corroborates the spectrophotometric findings and expands the range of techniques available for assessing the TPC. These results set the stage for future smartphone-based analytical methods in the field of food science, offering researchers and industry professionals a promising tool for swiftly and accurately measuring phenolic content. This method’s utility extends beyond TPC analysis in vegetable oils. It can effectively estimate antioxidant activity, determine certain phenolic groups such as anthocyanins, and assess tetraterpenoid pigments like carotenoids in plants, extracts, juices, and beverages. However, it is important to note that traditional methods may still have an advantage in dealing with complex sample matrices or requiring specific compound identification. Further studies are necessary for optimization and validation.

## Figures and Tables

**Table 1:** Specification of Huawei P30 Lite smartphone.

Specification	
Display	- Screen size: 6.15 inches - Screen color: 16.7 million colors, color saturation (NTSC): 96% (typical value) - Screen type: LTPS-TFT - Resolution: FHD 2312 × 1080 pixels (density 415 PPI) - Touch screen: multi-touch, support, maximum, up to 10 touch points during touch - Glass material: aluminosilicate glass - Screen-to-body ratio: 90.0%
Processor	- CPU model: Kirin 710 (Taiwan Semiconductor Manufacturing Company, Inc., Hsinchu, Taiwan) - CPU Kernel: Octa-core - CPU frequency: 4 × Cortex-A73 2.2 GHz (ARM Holdings, Cambridge, England, UK) + 4 × Cortex-A53 1.7 GHz (ARM Holdings, Cambridge, England, UK) - GPU model: Mali-G51 1 GHz (ARM Holdings, Cambridge, England, UK)
Operating system	- EMUI 9.0 (based on Android 9)
Main camera	- 48 MP (f/1.8 aperture) + 8 MP (f/1.8 aperture) + 2 MP (f/2.4 aperture) - Shooting modes: wide-angle lens, night, portrait, pro, slow motion, panorama, light painting, HDR, time lapse, 3D panorama, stickers, documents, ultra snapshots, smile capture, audio control, timer - Video recording: supports 4K video recording. EIS is supported - Photo resolution: 8000 × 6000 pixels - Video resolution: UHD 4K (Huawei Technologies Co., Ltd., Shenzhen, China)

**Table 2:** Changes in white and black are affected by the distance between a smartphone lens and the X-Rite ColorChecker color panel.

Distance, cm	Color	R	G	B
9	White	187 ± 1	185 ± 1	188 ± 1
	Black	20 ± 1	19 ± 1	20 ± 1
10	White	188 ± 1	186 ± 1	188 ± 1
	Black	19 ± 1	19 ± 1	20 ± 1
11	White	188 ± 1	187 ± 1	189 ± 1
	Black	18 ± 1	20 ± 1	19 ± 1
12	White	189 ± 1	189 ± 1	190 ± 1
	Black	17 ± 1	19 ± 1	18 ± 1
13	White	188 ± 1	189 ± 1	188 ± 1
	Black	18 ± 1	20 ± 1	19 ± 1
14	White	187 ± 1	188 ± 1	187 ± 1
	Black	18 ± 1	20 ± 1	19 ± 1
15	White	180 ± 1	180 ± 1	182 ± 1
	Black	19 ± 1	18 ± 1	19 ± 1

**Table 3:** Gallic acid *F*-test calculation at different calibration levels.

Indices	Gallic Acid Concentration (1–200 mg L^−1^)		Indices
*a*	0.00563	0.006	*b*
*a* error	0.00015	0.012	*b* error
*r* ^2^	0.993	0.033750898	*y* error
*F*	1375.24616	9	df
Regression sum of squares	1.56657465	0.010252108	Difference of sum of squares
*F*-test	3.7374 × 10^−11^		
**Gallic Acid Concentration (1–160 mg L^−1^)**			
*a*	0.00608	−0.002	*b*
*a* error	0.00007	0.004	*b* error
*r* ^2^	0.999	0.01139195	*y* error
*F*	6778.851302	8	df
Regression sum of squares	0.879735097	0.00103821	Difference of sum of squares
*F*-test	5.28141 × 10^−13^		

**Table 4:** Gallic acid calibration limits of detection (LOD) and quantification (LOQ).

Indices	Absorption
**Gallic acid, mg L^−1^**	
LOD	1.254
LOQ	3.801
**Measuring range, mg L^−1^**	
from	3.801
to	160

**Table 5:** Accuracy and repeatability of the method for analysis of total phenolic content in vegetable oil samples.

Sample (*n* = 10)	%RSD	Repeatability mg GAE 100 g^−1^		Accuracy mg GAE 100 g^−1^	
		Average	Max	Average	Max
Vegetable oils	0.67	0.0344	0.1414	0.0689	0.2828

## Data Availability

The original contributions presented in the study are included in the article, further inquiries can be directed to the corresponding authors. Python script can be found on the Project home page: https://github.com/SanitaVucane/Analysis-of-Smartphone-images-in-RGB-colour-system (accessed on 14 May 2024). Operating system(s): e.g., Platform independent, Programming language: e.g., Python, Other requirements: e.g., NumPy, Pandas, Matplotlib and SciPy, License: e.g., GNU GPL version 3., Any restrictions to use by non-academics: e.g., license needed.
